# Association between *AKT1* Gene Polymorphism rs2498794 and Smoking-Related Traits with reference to Cancer Susceptibility

**DOI:** 10.1155/2015/316829

**Published:** 2015-06-07

**Authors:** Daisuke Nishizawa, Shinya Kasai, Junko Hasegawa, Naomi Sato, Fumihiko Tanioka, Haruhiko Sugimura, Kazutaka Ikeda, Yoh Dobashi

**Affiliations:** ^1^Addictive Substance Project, Tokyo Metropolitan Institute of Medical Science, Tokyo 156-8506, Japan; ^2^Department of Clinical Nursing, Hamamatsu University School of Medicine, Hamamatsu 431-3192, Japan; ^3^Department of Tumor Pathology, Hamamatsu University School of Medicine, Hamamatsu 431-3192, Japan; ^4^Department of Pathology, Iwata City Hospital, Iwata 438-8550, Japan; ^5^Department of Pathology, Saitama Medical Center, Jichi Medical University, Saitama 330-8503, Japan

## Abstract

To clarify the potential role of variability within and around the *AKT1* gene in smoking behaviors, we performed a single-nucleotide polymorphism (SNP) analysis of the *AKT1* gene in an elderly Japanese cohort. Genotypes of the rs2498794 SNP, which is located in the fifth intron region of the *AKT1* gene, were marginally but significantly associated with smoking duration in the total 999 samples of former and current smokers. Interestingly, this SNP had a marginally significant association with individual cancer history (past and current), especially in groups with a shorter smoking duration (<44 years) and fewer cigarettes per day (≤20). These data suggest that the rs2498794 polymorphism of the *AKT1* gene is associated with a long smoking duration and may be involved in the predisposition to cancer when the smoking duration is short or the cigarettes per day is rate low.

## 1. Introduction

Akt (also called protein kinase B) is a serine-threonine kinase that was first identified in mice as the cellular homologue of the murine thymoma oncogene v-Akt [1, 2]. Three mammalian isoforms of its gene products have been identified—Akt1 (PKB*α*), Akt2 (PKB*β*), and Akt3 (PKB*γ*)—showing a broad tissue distribution [1–3]. Akt1 is the most ubiquitously expressed isoform. Although Akt2 and Akt3 are also ubiquitously expressed, Akt2 is expressed predominantly in insulin-responsive tissues, and Akt3 is expressed predominantly in the testes and brain [4]. Each Akt isoform is a downstream effector of the growth factor signaling pathway and functions as a mediator of the phosphoinositide-3 kinase (PI3K-Akt) pathway [5]. The stimuli that emanate from activated growth factor receptors activate this kinase cascade through PI3K and a second messenger, phosphatidylinositol (3,4,5)-trisphosphate, which then binds to Akt. Consequently, Akt is phosphorylated and activated by PI3K-dependent kinases 1 and 2 and activates various substrates, including mammalian target of rapamycin, Bad, Bax, Mdm2, and Foxo [2, 5, 6]. Akts phosphorylate and regulate various substrates that are involved in diverse cellular functions, including cell growth, survival, apoptosis, and metabolism, through the activation of translation [1–3, 7, 8]. Protein overexpression and activation and somatic aberrations of PI3K-Akt pathway genes have been commonly observed in a variety of malignancies, and this pathway has been extensively investigated as one of the critical mechanisms in tumorigenesis and as a target for cancer therapy [7, 9].

With regard to genetic aberrations,* AKT1* amplification has been reported in carcinomas of the lungs, stomach, breast, and prostate [2, 10, 11].* AKT2* gene amplification has been observed in carcinomas of the breast, ovaries, and pancreas and associated with a poor prognosis in several of these cancers [2, 11, 12].

Additionally, genetic variations of* AKT*s, such as single-nucleotide polymorphisms (SNPs), have also been well recognized to modulate gene function. These variations are associated with a predisposition to and determinant of clinical outcomes of endometrial and lung cancers [13–15].

Akt1 is also centrally involved in neuronal survival and plasticity [16].* AKT1* variations have been reported to be associated with Parkinson's disease, schizophrenia, methamphetamine use disorder, and bipolar disorder [16–21]. In light of the critical role of Akt in maintaining proper cellular function and tumorigenesis and/or tumor progression, the screening of* AKT* SNPs is important. Among the* AKT* genes, the present study focused on the* AKT1* gene, which is the most ubiquitously expressed and assumed to play central roles in various functions and pathologies.

With the goal of identifying allelic variants that significantly contribute to pathogenesis and smoking-related traits, global tests of associations were performed between each SNP of the* AKT1* gene and cancer predisposition and smoking behaviors.

## 2. Methods

### 2.1. Subjects

The participants in the initial analysis that explored possible associations between* AKT1* gene polymorphisms and the susceptibility to common cancers and smoking behavior included a total of 999 patients who presented at or were admitted to Iwata City Hospital in Japan. The inclusion criteria for this study were being Japanese, ambulatory, able to communicate orally, and 60 years of age or older. Numerous participants in this study had various smoking habits and completed a questionnaire that consisted of various questions about lifestyle, including alcohol consumption, smoking, diet, and cancer history [22, 23]. Peripheral blood samples were collected from these subjects for the gene analysis. The detailed demographic and clinical characteristics of the subjects, with a focus on cancer and smoking behaviors, are provided in Table 1. These data were used in the statistical analyses. Smoking duration (years), cigarettes smoked per day (CPD), and the product of these two (i.e., the Brinkman (smoking) index) were incorporated in the analysis for smoking behaviors.

The study protocol was approved by the Institutional Review Boards at Hamamatsu University School of Medicine (Hamamatsu, Japan) and the Tokyo Metropolitan Institute of Medical Science (Tokyo, Japan). All of the subjects provided informed, written consent for the genetics studies.

### 2.2. Genotyping

Genomic DNA was extracted from whole-blood samples using a QIAamp DNA BloodMaxi kit according to the manufacturer's instructions (Qiagen, Hamburg, Germany). The extracted DNA was dissolved in TE buffer (10 mM Tris-HCl and 1 mM ethylenediaminetetraacetic acid, pH 8.0) before use. The DNA concentration was adjusted to 100 ng/*μ*L for whole-genome genotyping and approximately 5–50 ng/*μ*L for genotyping the specific rs2498794 SNP using a NanoDrop ND-1000 Spectrophotometer (NanoDrop Technologies, Wilmington, DE, USA).

Briefly, whole-genome genotyping was performed using the Infinium assay II with an iScan system (Illumina, San Diego, CA, USA) according to the manufacturer's instructions. HumanCytoSNP-12 v2.0 (total markers: 301,232) BeadChips were used to genotype the 300 samples from the patients with clinical data for cancer and smoking history. The BeadChips included a number of probes that are specific to copy number variation markers, but most of the BeadChips were for SNP markers on the human autosome or sex chromosome. In the data-cleaning process, the samples with a genotype individual level call rate <0.95 were intended to be excluded from the analyses. Additionally, markers with a genotype call frequency <0.95 or “Cluster sep” (i.e., an index of genotype cluster separation) <0.1 were excluded from the subsequent association study. Markers were not excluded based on the heterozygosity rates and results of Hardy-Weinberg equilibrium (HWE) tests for the whole-genome genotyping data, but the HWE tests were conducted for the selected individual SNPs for association analyses. Tests for population substructure and relatedness were not conducted because it was assumed that all of the subjects were unrelated and genetically homogeneous Japanese, mostly living in the Kanto or Tokai area. As a result, a total of 291,523 SNP markers survived the filtration process and were used for the dataset of the association analyses.

To genotype the rs2498794 SNP using a total of 700 DNA samples in the subsequent association study after an initial exploratory association study, the TaqMan allelic discrimination assay (Life Technologies, Carlsbad, CA, USA) was basically adopted. To perform the TaqMan allelic discrimination assay with a LightCycler 480 (Roche Diagnostics, Basel, Switzerland), TaqMan SNP Genotyping Assays (Life Technologies) were used that contained sequence-specific forward and reverse primers to amplify the polymorphic sequence and two probes labeled with VIC and FAM dye to detect both alleles of the candidate Tag SNP, rs2498794 (assay ID: C____193159_10). Real-time polymerase chain reaction was performed in a final volume of 10 *μ*L that contained 2× LightCycler 480 Probes Master (Roche Diagnostics), 40× TaqMan Genotyping Assays, 5–50 ng genomic DNA as the template, and up to 10 *μ*L H_2_O (Roche Diagnostics). The thermal conditions were the following: 95°C for 10 min, followed by 45 cycles of 95°C for 10 s and 60°C for 60 s, with final cooling at 50°C for 30 s. Afterward, endpoint fluorescence was measured for each sample well, and each genotype was determined based on the presence or absence of each type of fluorescence.

### 2.3. Linkage Disequilibrium Analysis

To initially analyze SNPs within and around the* AKT1* gene region, genotype data for approximately 300,000 SNP markers that resulted from whole-genome genotyping with the patient samples with clinical data for cancer and smoking history were basically used, and the genotype data for all of the SNPs with* AKT1* gene annotation were extracted for a total of 300 samples. The minor allele threshold for the SNP selection was set at 0.001, which indicates the inclusion of at least one minor allele carrier in the 300 samples. As a result, the rs28546406 SNP was dropped based on the minor allele frequency criterion.

Of the seven available SNPs with minor allele frequencies above 0.001 that were located within the exon and intron regions and approximately within the 10 kbp 5′- and 3′-flanking regions of the* AKT1*, SNPs for the association studies were selected based on standard tagging strategies regardless of the functionality of the SNPs [24–26]. To identify relationships between the SNPs that were used in the study and reduce the burden of tests because some tests were not independent, a linkage disequilibrium (LD) analysis was performed for 300 samples using Haploview v. 4.1 [27]. To estimate the LD strength between the SNPs, the commonly used *D*′ and *r* ^2^ values were pairwise-calculated using the genotype dataset of each of the seven SNPs. Linkage disequilibrium blocks were defined among the SNPs with minor allele frequencies above 0.05 that showed “strong LD,” based on the default algorithm of Gabriel et al. [28], in which the upper and lower 95% confidence limits on *D*′ for strong LD were set at 0.98 and 0.7, respectively. Tag SNPs in the LD block were consequently determined using the Tagger software package with default settings, which is incorporated in Haploview and has been detailed in a previous report [26]. The Tag SNPs in the LD block and common SNPs outside the block with minor allele frequencies above 0.05 were selected for the association analyses.

### 2.4. Statistical Analysis

A total of 300 subjects were used for the initial LD and association analyses. For all of the genotype data that were used in these analyses, the distributions were checked in the entire cohort using the *χ* ^2^ test, and the absence of significant deviation from the theoretical distribution that was expected from Hardy-Weinberg equilibrium was confirmed. Prior to the analyses, the subjects were divided into two subgroups based on the presence and absence of common cancers (present and past illness), in addition to dividing the subjects into three smoking subgroups: current smokers, exsmokers, and never-smokers (Table 1). To explore the associations between the clinical characteristics of the total of 999 subjects, the *χ* ^2^ test or Mann-Whitney test was performed overall, and statistical significance was set to *P* < 0.05. To explore the associations between the SNPs and phenotypes related to smoking and cancer in the initial 300 subjects, the *χ* ^2^ test was performed overall, and SNPs that showed *P* < 0.05 in the analysis were considered nominally significant and selected for further analysis. In the following confirmatory stage of the analysis in the remaining 699 subjects, the *χ* ^2^ test was again performed overall to corroborate the association that was observed in the exploratory stage of the analysis. Analyses of interactions between genotypes of the candidate SNP and smoking-related phenotypes, such as smoking history, smoking period, CPD, and the Brinkman index, were conducted with the statistical significance set to *P* < 0.05 after dividing the entire sample of subjects with available smoking-related phenotype data into two groups based on categorical phenotypes or higher/lower values of quantitative phenotypes compared with each median value, considering the classifications of previous reports and expected correlations among the phenotypic values [29, 30]. Statistical corrections for multiple tests, such as Bonferroni adjustments on the multiple parameters analyzed, were not performed in the present exploratory study as a whole because it would be too conservative for genetic association studies [31], meaning that the likelihood of type II errors is increased by Bonferroni adjustments, and truly important differences could be deemed nonsignificant [32]. All of the statistical analyses were performed using SPSS 18.0J software (International Business Machines Corporation, Armonk, NY, USA).

Statistical power analyses were performed using G^*^Power 3.1.3 [33]. Power analyses for the *χ* ^2^ tests revealed that the expected power (1 minus type II error probability) was 99.9% and 100% for Cohen's conventional “medium” effect size of 0.30 [34] when the degrees of freedom and type I error probability were set at 1 and 0.05, respectively, and the sample sizes were 300 and 699, respectively, corresponding to the sample sizes of the exploratory analyses and subsequent confirmatory analyses in the present study. However, for the same type I error probability and sample sizes of 300 and 699, the expected power decreased to 41.0% and 75.3%, respectively, when Cohen's conventional “small” effect size was 0.10. Conversely, the estimated effect sizes were 0.1617 and 0.1060 for the same type I error probability and sample sizes of 300 and 699, respectively, to achieve 80% power. Therefore, a single analysis in the present study was expected to detect true associations with the phenotype with 80% statistical power for effect sizes from large to moderately small but not very small.

## 3. Results

We explored the contribution of the SNPs in and around* AKT1 *SNPs to various smoking traits and individual cancer history in the initial 300 subjects, followed by confirmatory analyses in the remaining 699 subjects. In the analyses of the clinical data prior to association analyses concerning SNPs in a total of 999 subjects, significant differences were found in sex (*χ* ^2^ = 9.876, *P* = 0.0017), height (*U* = 71176.500, *P* = 0.0104), CPD (*U* = 20187.000, *P* = 0.0304), and the Brinkman index (*U* = 19746.500, *P* = 0.0170) between individuals with a current and past cancer history and those without any cancer history. Subjects who were male, were taller, had a greater CPD, and had a higher Brinkman index were more susceptible to any cancer compared with controls (i.e., higher in risk of cancer; Table 2). In the list of cancer history (Table 1), 95 of 105 subjects with a current history of cancer and 127 of 136 subjects with a past history of cancer were smoking-related cases according to Surgeon General's report [35], and the list of cancers that are associated with smoking is becoming longer [36]. In the analysis that focused only on smoking-related cancers, in which cancers of the uterus, thyroid, biliary tract (gallbladder/bile duct), ovaries, bones, and other organs (Table 1) were excluded from the analysis, and significant differences were found in smoking history (*χ* ^2^ = 7.244, *P* = 0.0071), sex (*χ* ^2^ = 17.325, *P* < 0.0001), height (*U* = 64641.500, *P* = 0.0006), CPD (*U* = 20086.000, *P* = 0.0316), and the Brinkman index (*U* = 19732.500, *P* = 0.0209) between individuals with a current and past history of cancer and those without any history of cancer. A similar analysis was conducted after stratifying the data by sex, which revealed significant differences in smoking history (male: *χ* ^2^ = 0.146, *P* = 0.7020; female: *χ* ^2^ = 5.603, *P* = 0.0179) and age (male:* U* = 28378.000, *P* = 0.0256; female:* U* = 9833.000, *P* = 0.9731) between the cancer and control subjects. In the analysis that focused only on the smoking-related cancers mentioned above, significant differences were again found in smoking history (male: *χ* ^2^ = 0.284, *P* = 0.5942; female: *χ* ^2^ = 4.483, *P* = 0.0342) and age (male:* U* = 28071.000, *P* = 0.0244; female:* U* = 8682.000, *P* = 0.6950) between the cancer and control subjects. Female subjects without a smoking history and male subjects who were older were more susceptible to cancer compared with controls (i.e., they had a higher risk of cancer; details not shown).

**Figure 1:**
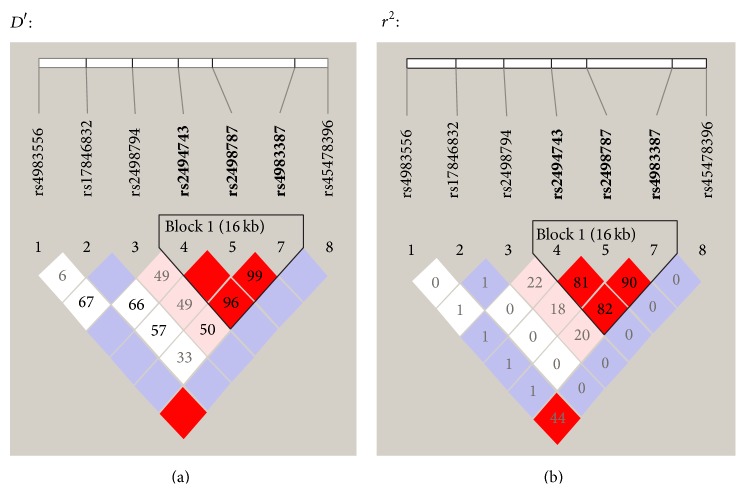
State of linkage disequilibrium (LD) between the SNPs within the exon and intron regions and approximately within the 10 kbp 5′- and 3′-flanking regions of the* AKT1* gene on chromosome 14, based on the genotype data of the 300 samples used in the initial exploratory association analysis. Numbers in squares in which two SNPs face represent the percentage of *D*′ and *r* ^2^ values calculated from the genotype data of the SNPs in (a) and (b), respectively. Blank squares represent *D*′ = 1 or *r* ^2^ = 1.

**Figure 2:**
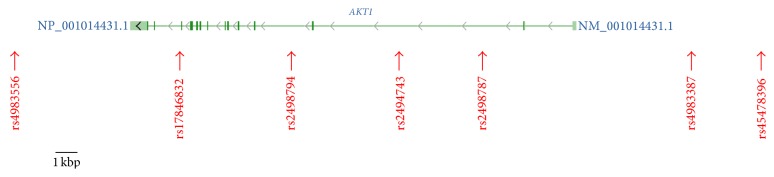
Schematic structure of the human* AKT1* gene (gene accession number: NM_001014431.1) and the location of the seven SNPs that were used in the association analyses, based on the NCBI database (http://www.ncbi.nlm.nih.gov/gene/, accessed May 13, 2015). The gene is illustrated between the 3′- and 5′-flanking regions, corresponding to left and right sides, respectively. Green boxes and a line connecting them represent the exon and intron, respectively.

After whole-genome genotyping, an LD analysis was initially conducted using the genotype data from 300 samples among a total of 999 samples (Table 1). An LD block was observed among the seven SNPs with minor allele frequencies above 0.001 that were located within and around the* AKT1* gene region, and all three SNPs in the block were selected as Tag SNPs in the LD block (Figure 1). The schematic structure of the gene and location of the SNPs are illustrated in Figure 2. Only one SNP, rs2498794, was located outside the block, with minor allele frequencies above 0.05. Therefore, a total of four common SNPs (rs2498794, rs2494743, rs2498787, and rs4983387) were selected for the association analyses. Of these SNPs, only one SNP, rs2498794, was found to be nominally significant (*P* < 0.05) in the initial exploratory association analysis between the SNPs and smoking or cancer-related phenotypes (Table 3). This SNP was significantly associated with cancer status (present illness) in the recessive model for the minor T allele, in which the T/T genotype of this SNP was associated with an increased risk of cancer. A further analysis of the remaining 699 samples to confirm the association that was observed in the exploratory analysis was conducted for this SNP. However, no significant association was found for this sample set, and the association between the rs2498794 SNP and cancer status (present illness) was not significant, even in the combined 999-sample set (Table 4). Although a significant association was found between this SNP and cancer status (either present or past history combined) in the confirmatory analysis with decreased cancer risk in the T/T genotype of this SNP, the association was not significant in the combined sample set (Table 4), suggesting that the influence of this SNP on the susceptibility to lifetime cancer risk may not be substantial. Among the other phenotypic traits, a significant association was found between the rs2498794 SNP and smoking duration (years) in the combined sample set (Table 4). Homozygous carriers of the minor T allele in this SNP had longer smoking histories compared with noncarriers, suggesting that this SNP may affect smoking behavior, leading to a prolonged period of smoking in T/T carriers of this SNP.

The observed association between the rs2498794 SNP and smoking duration in the total of 999 samples could be of interest, but we could not draw definitive conclusions about such a relationship because an association was not found in either the initial exploratory analyses or subsequent confirmatory analyses. We further examined interactive effects between this SNP and phenotypes in the overall subjects with available smoking-related phenotype data, such as smoking history, smoking period, CPD, and the Brinkman index. We compared genotype data between the presence and absence of cancer history after dividing the subjects into two groups based on long/short smoking histories and higher/lower values in quantitative variables compared with each median value. This analysis resulted in significant associations between the rs2498794 SNP and cancer status (present or past history, combined) only in groups with a shorter smoking duration (<44 years) and lower CPD (≤20). Homozygous carriers of the minor T allele in this SNP were fewer in cancer subjects than in controls when the smoking duration was short and CPD rate was low (Table 5), suggesting that the T/T genotype of this SNP may be related to lower susceptibility to cancer (i.e., this genotype could be associated with a lower risk of cancer only when the smoking duration is short or CPD rate is low).

## 4. Discussion

The PI3K/Akt pathway fulfills an important role in cell metabolism, proliferation, apoptosis, and metastasis [2, 5]. As one of the key components of this pathway, somatic mutations of* AKTs* have also been reported. An activating mutation in* AKT1* (E17K), which results in the growth factor-independent membrane translocation of Akt and increased phosphorylation levels [37], was identified in various types of cancers, including melanoma and breast, esophageal, colorectal, endometrial, ovarian, and nonsmall cell lung cancers [38]. Moreover, an identical mutation in* AKT2* was found to cause its membrane localization and the insulin-independent membrane localization of the GLUT4 glucose transporter and subsequent hypoglycemia [39].* AKT1* gene variations, including haplotypic variations, have been reported to be associated with various cellular pathological and biological phenotypes, such as resistance to apoptosis in Epstein-Barr virus-transformed lymphocyte cells [40], and the cellular response to DNA damage [40]. Furthermore, associations with human behavior, including psychiatric diseases, have been reported [18, 20]. There is one report of an association with endometrial cancer susceptibility [15]. The present study examined seven* AKT1* gene variations in humans (to the extent that they were on the HumanCytoSNP-12 v2.0 BeadChip) and explored associations between these variations and outcomes in cancer and smoking behavior that could be related to each other. The rs2498794 SNP was potently associated with smoking duration. Homozygous carriers of the T allele of this SNP had prolonged smoking durations compared with noncarriers. The subsequent interactive association study indicated that this SNP was also associated with the susceptibility to cancer only in subgroups with a shorter smoking duration or lower CPD, in which homozygous carriers of the T allele of this SNP had a lower probability of predisposition to cancer (i.e., a decreased risk of cancer). The reason for this may lie in the fact that smoking for longer periods of time and a higher CPD are well known general risk factors for cancer, particularly lung cancer, regardless of polymorphisms [41]. Although future studies should attempt to replicate the present results, the present study demonstrated the possibility that the rs2498794 SNP may be marginally associated with both smoking duration and cancer risk when the smoking duration is short and CPD rate is low.

The candidate SNP that was selected in the present study, rs2498794, is located in the fifth intron region of the* AKT1* gene. To date, associations between variations in this site with pathological states, clinical features, or overt diseases have not been reported. This SNP did not show strong LD (e.g., *r* ^2^ ≥ 0.8) with other SNPs within and around the* AKT1* gene region. Additionally, this SNP may not affect splicing of the* AKT1* gene or regulatory potential scores according to SNP Function Prediction (FuncPred) in the SNPinfo Web Server, which compiles information on SNP function predictions and ethnicity-specific allele frequencies (http://snpinfo.niehs.nih.gov/snpinfo/snpfunc.htm, accessed May 13, 2015). Although these results appear to reflect a relatively low possibility that phenotypic alterations that are related to the rs2498794 SNP are attributable to alterations in the function or expression of Akt1 that are caused by this SNP or other SNPs that are in strong LD with this SNP, future studies will clarify possible alterations in function or expression that are related to such* AKT1* SNPs. The rs2494731 SNP, which is located in the intron region (similar to the rs2498794 SNP), is annotated as an SNP that shows moderately strong LD (e.g., *r* ^2^ ≥ 0.7) with the rs2498794 SNP based on the SNPinfo Web Server and is reportedly associated with the risk of suicidal behavior in bipolar patients [42]. In addition to the present results that showed a marginally significant association between the rs2498794 SNP and smoking duration in the combined 999 samples, the rs2498794 SNP may also be associated with neurobiological mechanisms that underlie psychiatric disorders or related phenotypes. Future studies are needed to clarify the underlying mechanisms by which cancer or smoking-related phenotypes are modulated by this SNP. Our findings may provide novel insights for future investigations.

Although the observed associations in the present study might be restricted to the Japanese population and the underlying mechanisms remain to be fully elucidated, the present results suggest that the rs2498794 SNP may be a marker that predicts prolonged smoking duration and a cornerstone for future association and functional studies that focus on this SNP.

## Figures and Tables

**Table 1:** Demographic data of patients.

	*n*	Minimum	Maximum	Mean	SD	Median
All subjects	999					
Male	612					
Female	387					
Age (years)	999	60	94	73.58	5.86	73.00
Height (cm)	996	130	180	156.86	8.69	158.00
Weight (kg)	996	30	101	54.75	9.98	54.00
Smoking status	999					
Current smokers	130					
Exsmokers	392					
Never-smokers	477					
Smoking behaviors	522					
Smoking period (year)	520	1.00	68.00	40.50	14.40	44.00
CPD^a^	520	1.00	100.00	21.44	13.42	20.00
Brinkman (smoking) index	519	9.00	3480.00	853.32	588.64	700.00
Cancer status (present history)	999					
Present	89					
Absent	874					
Unknown	36					
Cancer status (past history)	999					
Present	128					
Absent	848					
Unknown	23					
Cancer sites (present/past history)	105/136					
Oral mucosa/tongue	2/2					
Upper digestive tract	15/36					
Lower digestive tract	8/23					
Liver	6/4					
Biliary tract (gallbladder/bile duct)	2/1					
Pancreas	2/2					
Breast	6/10					
Lung	11/12					
Hematolymphoid system	6/2					
Ovary	1/1					
Uterus	3/5					
Kidney	1/8					
Bladder	2/7					
Prostate	35/20					
Thyroid	1/1					
Skin	1/1					
Bone	1/0					
Others	2/1					

^a^Cigarettes smoked per day.

**Table 2:** Comparisons of clinical data between presence/absence of cancer history.

	*n*	Minimum	Maximum	Mean	SD	Median	Statistic^a^	*P*	Direction of association
Sex	962						9.88	0.00167^*∗*^	Higher cancer risk in males
Cancer (male/female)	148/63								
Control (male/female)	437/314								
Age (years)	970						74228.50	0.06514	
Cancer	214	64	88	74.12	5.73	74.00			
Control	756	60	94	73.43	5.89	73.00			
Height (cm)	968						71176.50	0.01035^*∗*^	Higher height in cancer subjects
Cancer	213	140	175	158.20	7.84	160.00			
Control	755	130	180	156.45	8.92	157.00			
Weight (kg)	967						82386.00	0.56215	
Cancer	213	30	101	54.53	10.19	54.00			
Control	754	30	90	54.77	9.90	54.00			
Smoking history	971						3.10	0.07831	
Cancer (ever-smokers/never-smokers)	92/122								
Control (ever-smokers/never-smokers)	377/380								
Smoking period (year)	501						20624.50	0.07276	
Cancer	122	10.00	64.00	42.45	13.63	46.00			
Control	379	1.00	68.00	39.71	14.64	44.00			
CPD^b^	501						20187.00	0.03041^*∗*^	Higher CPD in cancer subjects
Cancer	122	2.50	80.00	23.75	15.10	20.00			
Control	379	1.00	100.00	20.77	12.90	20.00			
Brinkman (smoking) index (BI)	500						19746.50	0.01700^*∗*^	Higher BI in cancer subjects
Cancer	122	100.00	3360.00	1001.64	708.25	860.00			
Control	378	9.00	3480.00	809.38	547.47	680.00			

^a^ *U* value for Mann-Whitney test and *χ* ^2^ value for *χ* ^2^ test.

^b^Cigarettes smoked per day.

^*∗*^ *P* < 0.05.

**Table 3:** Results of exploratory association analysis between the common *AKT1* SNPs and smoking or cancer-related phenotypes.

SNP	CHR^a^	Position^b^	Location	MAF^c^	Major/minor alleles	*P*values for exploratory association analysis in genotypic/dominant/recessive models for each minor allele					
						Cancer status (present illness)	Cancer status (present or past illnesses)	Smoking history	Smoking period (year)	CPD^d^	Brinkman (smoking) index
rs4983556	14	104302924	3′-flanking	0.015	T/G	—	—	—	—	—	—
rs17846832	14	104309681	intron	0.0233	C/T	—	—	—	—	—	—
rs2498794	14	104316296	intron	0.4167	C/T	0.120/0.851/0.043^*∗*^	0.495/0.293/0.412	0.464/0.809/0.217	0.420/0.694/0.265	0.479/0.344/0.622	0.468/0.445/0.462
rs2494743	14	104322765	intron	0.4381	C/T	0.586/0.351/0.472	0.509/0.886/0.255	0.632/0.340/0.822	0.997/0.980/0.947	0.993/0.962/0.906	0.983/0.860/0.994
rs2498787	14	104327626	intron	0.4897	A/G	0.903/0.736/0.692	0.613/0.334/0.600	0.984/0.929/0.906	0.871/0.931/0.645	0.963/0.896/0.787	0.751/0.455/0.720
rs28546406	14	104333890	5′-flanking	0	G/G	—	—	—	—	—	—
rs4983387	14	104339273	5′-flanking	0.47	G/A	0.732/0.890/0.490	0.760/0.459/0.824	0.561/0.780/0.373	0.963/0.826/0.926	0.835/0.659/0.604	0.708/0.488/0.520
rs45478396	14	104343869	5′-flanking	0.0067	C/T	—	—	—	—	—	—

^a^Chromosome number.

^b^Chromosomal position (bp).

^c^Minor allele frequency.

^d^Cigarettes smoked per day.

^*∗*^Significant association between the minor T allele of the SNP and cancer status (present illness) in the recessive model (*P* < 0.05).

**Table 4:** Results of confirmatory association analysis between the rs2498794 SNP and smoking or cancer-related phenotypes.

SNP	Stage	*P*values for confirmatory and combined association analyses in genotypic/dominant/recessive models for each minor allele					
		Cancer status (present illness)	Cancer status (present or past illnesses)	Smoking history	Smoking period (year)	CPD^a^	Brinkman (smoking) index
rs2498794	confirmatory	0.208/0.453/0.077	0.075/0.903/0.038^*∗*^	0.552/0.892/0.336	0.143/0.743/0.092	0.912/0.890/0.744	0.141/0.733/0.092
rs2498794	combined	0.910/0.665/0.890	0.205/0.482/0.190	0.901/0.824/0.763	0.068/0.641/0.048^†^	0.610/0.529/0.610	0.073/0.486/0.070

^a^Cigarettes smoked per day.

^*∗*^Significant association between the minor T allele of the SNP and noncancer status (present or past illness) in the recessive model (*P* < 0.05).

^†^Significant association between the minor T allele of the SNP and longer periods of smoking history in the recessive model (*P* < 0.05).

**Table 5:** Comparisons of genotype data between presence/absence of cancer history stratified by smoking-related phenotypes.

	Genotype			Genotypic model		Dominant model			Recessive model		
	TT	TC	CC	*χ* ^2^	*P*	OR (95% CI)^a^	*χ* ^2^	*P*	OR (95% CI)^a^	*χ* ^2^	*P*
*Smoking history *											
Ever-smokers											
Cancer	17	65	40	3.281788	0.19381	1.017 (0.659–1.570)	0.005743	0.93959	0.617 (0.349–1.090)	2.805891	0.09392
Control	79	175	126								
Never-smokers											
Cancer	16	50	25	1.234473	0.53943	1.299 (0.782–2.159)	1.022436	0.31194	0.966 (0.530–1.761)	0.012551	0.91080
Control	68	184	124								
*Smoking period (year) *											
Long (≥44)											
Cancer	13	31	24	0.392140	0.82195	1.226 (0.635–2.365)	0.181513	0.67008	1.226 (0.635–2.365)	0.341154	0.55916
Control	43	86	62								
Short (<44)											
Cancer	4	34	16	5.917681	0.05188	1.226 (0.635–2.365)	0.369160	0.54346	0.338 (0.115–0.996)	4.191842	0.04062^*∗*^
Control	36	88	64								
*CPD* ^b^											
High (>20)											
Cancer	9	19	12	1.526609	0.46612	1.642 (0.743–3.630)	1.514011	0.21853	1.194 (0.484–2.946)	0.147578	0.70086
Control	18	36	38								
Low (≤20)											
Cancer	8	46	28	5.512995	0.06351	0.849 (0.504–1.428)	0.382380	0.53633	0.402 (0.184–0.880)	5.491155	0.01911^†^
Control	61	139	88								
*Brinkman (smoking) index *											
High (>700)											
Cancer	12	33	24	1.322869	0.51611	0.961 (0.536–1.723)	0.017720	0.89410	0.671 (0.330–1.365)	1.223659	0.26864
Control	43	76	61								
Low (≤700)											
Cancer	5	32	16	3.103819	0.21184	1.130 (0.586–2.181)	0.133278	0.71506	0.469 (0.174–1.261)	2.341073	0.12600
Control	36	97	65								

^a^Odds ratio (95% confidence interval).

^b^Cigarettes smoked per day.

^*∗*^Significant association between the minor T allele of the SNP and noncancer status (present or past illness) in the recessive model (*P* < 0.05) in subjects whose smoking duration is short.

^†^Significant association between the minor T allele of the SNP and noncancer status (present or past illness) in the recessive model (*P* < 0.05) in subjects whose CPD rate is low.
